# Universal nuclear focusing of confined electron spins

**DOI:** 10.1038/s41467-019-08882-y

**Published:** 2019-03-07

**Authors:** Sergej Markmann, Christian Reichl, Werner Wegscheider, Gian Salis

**Affiliations:** 1 IBM Research-Zurich, Säumerstrasse 4, 8803 Rüschlikon, Switzerland; 2 Solid State Physics Laboratory, ETH Zurich, 8093 Zurich, Switzerland

## Abstract

For spin-based quantum computation in semiconductors, dephasing of electron spins by a fluctuating background of nuclear spins is a main obstacle. Here we show that this nuclear background can be precisely controlled in generic quantum dots by periodically exciting electron spins. We demonstrate this universal phenomenon in many-electron GaAs/AlGaAs quantum dot ensembles using optical pump-probe spectroscopy. A feedback mechanism between the electron spin polarization and the nuclear system focuses the electron spin precession frequency into discrete spin modes. Employing such control of nuclear spin polarization, the electron spin lifetime within individual dots can surpass the limit of nuclear background fluctuations, thus substantially enhancing the spin coherence time. This opens the door to achieve long electron spin coherence times also in lithographically defined many-electron systems that can be controlled in shape, size and position.

## Abstract (web summary)

One of the challenges in maintaining quantum coherence in semiconductor devices is coping with the fluctuating magnetic field generated by nearby nuclear spins. Here the authors explore the functioning of a feedback mechanism between electron and nuclear spins that extends the coherence time of a many-electron quantum dot.

## Introduction

Semiconductor quantum dots (QDs) exhibit tunable atomic-like electronic states^1^. For this reason, QDs are referred to as artificial atoms and may serve as hosts for spin quantum bits (qubits), the main building block for spin-based quantum computation^2^. One of the core problems of spin qubits is an undesired hyperfine interaction of electron or hole spins with the nuclear environment of the semiconductor host material. This issue is known as the central spin problem^3–6^. Even though the size of a self-assembled QD is few tens of nm, the confined electrons interact with *n* = 10^4^–10^6^ nuclear spins^7^. In this way the electron or hole spins experience an effective magnetic field *B*_n_ (Overhauser field), which arises from the averaged nuclear magnetic moments. Due to the statistical nature of the nuclear polarization, the Overhauser field fluctuates (on a scale ${\mathrm{\Delta }}B_{\mathrm{n}} \propto 1{\mathrm{/}}\sqrt n$), thus giving rise to electron spin dephasing. Reduction of random nuclear background has been successfully demonstrated utilizing spin echo techniques in electrostatically defined QDs^8–12^ as well as in self-assembled QDs^13–15^ by electrical and optical means, respectively. Control of nuclear field fluctuations has also been demonstrated using continuous-wave laser excitation in self-assembled QDs^16–18^. Furthermore, it has been shown that electron spin decoherence, arising from nuclear spin fluctuations, can be drastically reduced in inhomogenously broadened QD ensembles. This is realized by a self-synchronization of the electron spin precession with the repetition rate of a spin-exciting laser pulse train^19^ which was termed spin mode-locking. Spin mode-locking offers the prospect to overcome hyperfine-induced spin dephasing but up to now has only been observed in singly charged self-assembled QDs and recently in ZnSe:F donor-bound electrons^20^. Different explanations for how electron spin precession is synchronized to the repetition rate of spin excitation have been proposed^21–23^, but the details are not well understood yet.

Here we report that spin mode-locking is a universal phenomenon that also occurs in many-electron GaAs/AlGaAs QDs of variable diameters of up to 1800 nm. From optical pump-probe measurements, we find that mode-locking is absent for depolarized nuclear spins and emerges slowly (time scale of seconds) once the laser excitation pulse train is turned on. This suggests that spin mode-locking arises from dynamical nuclear polarization (DNP). We find that the mode-locking can be changed into an anti-mode-locking if the energy of the pump pulses is decreased below the absorption edge of the dots. This suggests that DNP is caused by the optical Stark effect where the pump pulses rotate transverse spin components into a direction parallel or antiparallel to the magnetic field^20,24,25^. In addition, we develop a model based on saturation of spin polarization that predicts spin mode-locking in an oblique magnetic field configuration. In that model, the resulting DNP decreases sharply if the laser repetition period matches an integer multiple of the spin precession period, thus providing a feed-back mechanism that drives nuclear polarization to a precise value. We test the model in an oblique magnetic field configuration and find either spin mode-locking or anti-mode-locking, depending on the pump wavelength, indicating that such a mechanism—if present—is overshadowed by the optical Stark effect. The observation of spin mode-locking in lithographically defined many-electron dots opens up a playground for spin manipulation and control in material systems where shape, spacing and positioning of the dots can be perfectly controlled. Importantly, by these mechanisms, the nuclear spin polarization becomes locked within a distribution that can be much narrower than the typical low-frequency fluctuations of nuclear spins in QDs, enabling long spin lifetimes in systems with nuclear background noise. Periodical excitation of the electron spins as a preparation step before starting a series of quantum operations could be a much simpler technique to enhance coherence times compared to dynamical decoupling schemes using spin echo pulses.

## Results

### Spin mode-locking

**Fig. 1:**
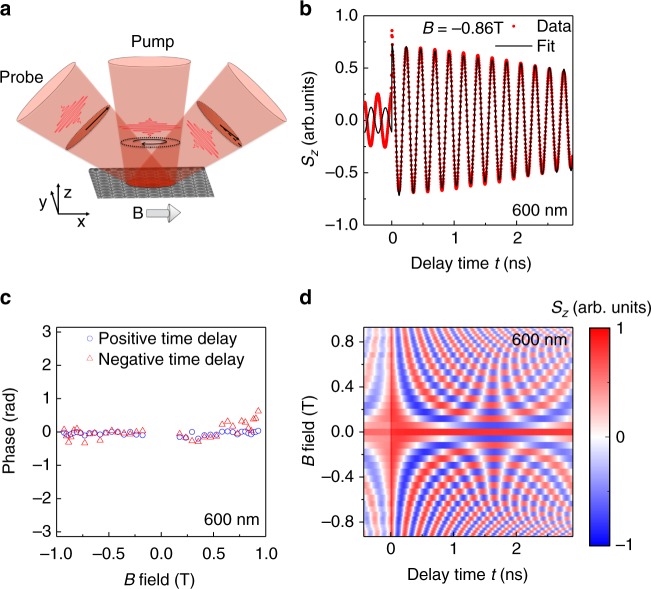
Time-resolved spin dynamics in dot arrays. **a** Sketch of time-resolved Kerr rotation measurement of spin component *S*_*z*_. **b** Recorded *S*_*z*_(*t*) at a fixed external magnetic field of a 600 nm dot array. **c** Extracted phase of the oscillation *S*_*z*_(*t*) at positive zero-delay (${{t}}_0^ +$) and at negative zero-delay (${{t}}_0^ -$) of a 600 nm dot array. **d** Recorded *S*_*z*_(*t*) as a function of external magnetic field *B* for 600 nm dot size array. The values of *S*_*z*_(*t*) are color coded and normalized to ±1

Samples are obtained from an n-doped GaAs/AlGaAs quantum well grown by molecular beam epitaxy. Arrays of disk-shaped QDs with diameters between 400 and 1800 nm are defined by e-beam lithography and dry etching (see Method section). Each dot contains between hundreds and several thousands of electrons depending on the dot size, as estimated from the quantum well carrier density (see Supplementary Note 1). We use time-resolved Kerr rotation measurements^26^ to study the electron spin dynamics of a QD ensemble. The spin polarization in the ensemble is excited with optical pump pulses propagating along the *z*-direction, perpendicular to the sample (*x*−*y*) plane. An external magnetic field **B** is applied along the *x*-direction. The circularly polarized pump pulses generate an electron spin polarization along the *z-*direction, which subsequently precesses about **B** (Fig. 1a). The spin polarization component *S*_*z*_ is measured by a time-delayed linearly polarized probe pulse via the magneto-optical Kerr effect. The picosecond-long pulses arrive with a repetition period $\tau _{\mathrm{r}}$ of 12.5 ns. The helicity of the pump pulses is modulated between *σ*^+^ and *σ*^−^ at a frequency of 50 kHz using a photoelastic modulator, thereby alternating between spin excitation along and against the *z* direction, facilitating lock-in detection of the Kerr signal.

Figure 1b shows a time-resolved Kerr signal (red points) taken on an ensemble of 600 nm large dots and with *B* = −0.86 T. The signal is proportional to the electron spin component *S*_*z*_. At zero-delay time *t*, a new pump pulse excites spin polarization that subsequently precesses in the external magnetic field, together with spin polarization that persists from the previous pump pulses. For increasing positive time delays, the precession amplitude decays. Such a decay is associated with two contributions. The first one is due to the electron spin lifetime ($\tau$) of a single dot and the second one arises from the inhomogeneous frequency broadening of the dot-ensemble ($\tau _{{\mathrm{inh}}}$). An exponential fit of the precession amplitude (black line in Fig. 1b) for *t* between 0 and 2.8 ns yields an effective spin lifetime of $\tau ^ \ast = 6.8\,{\mathrm{ns}}$, significantly enhanced in comparison to the measured time in the unstructured two-dimensional electron gas (0.5 ns), but smaller than the laser repetition period $\tau _{\mathrm{r}}$. We expect $\tau ^ \ast$ to be limited by $\tau _{{\mathrm{inh}}}$, resulting from a spreading of the g-factor distribution in the dot-ensemble. The single-dot lifetime $\tau$ is affected by dephasing from spin-orbit coupling, which is drastically suppressed^27–29^ as compared to a 2D electron gas, as has been recently shown also in wires^30,31^. Hyperfine interaction with fluctuating nuclear spin polarization becomes an additional contribution to spin dephasing for small dot sizes and scales reciprocally with the dot diameter.

It has been observed in ensembles of self-assembled QDs^21^ that the distribution of precession frequencies develops into a comb-like spectrum with the teeth of the comb sitting at frequencies that are integer multiples of the laser repetition rate. In such a mode-locked system, the spin precession phase immediately before ($t = t_0^ -$) and after ($t = t_0^ +$) the pump pulse arrival is identical. In addition, if more than one mode participates ($\tau > \tau _{\mathrm{r}} > \tau _{{\mathrm{inh}}}$), the spin polarization reemerges before a next pump pulse arrives. The measured *S*_*z*_ in Fig. 1b at negative time delays (equal to positive delays between 12.07 and 12.5 ns) ends with a positive maximum at $t = t_0^ -$, i.e. with the same phase as the spin polarization that precesses after $t = t_0^ +$. This equal phase is a strong indication of mode-locking. For an unlocked system, the phase at $t = t_0^ -$ should increase linearly with *B*. We have repeated measurements similar to the one shown in Fig. 1b at varying *B*. Obtained results are shown in Fig. 1d as a 2D-map. The two axes represent *B* and *t*, respectively. Over the full field range, we find a positive spin orientation at $t_0^ -$. From fits of the Kerr signal at $t_0^ +$ and $t_0^ -$, we extract the respective spin precession phases. At $t_0^ -$, we find a constant phase close to zero irrespective of *B*, clearly demonstrating spin mode-locking. We have omitted data points in Fig. 1c close to *B* = 0 since the determination of the phase is difficult if the spin precession period exceeds the experimentally available range of time delay. Spin mode-locking is consistently observed for all dot sizes between 400 and 1800 nm (see Supplementary Note 2).

Spin mode -locking as previously observed in singly charged self-assembled QDs^19^ has been explained by different models. Three of them^21–23^ rely on the specific property of trion excitation where the spin polarization of the electron added by the pump pulse depends on the orientation of the resident electron spin before the pump pulse^32,33^. This is due to the Pauli principle that requires the excitation of an antiparallel spin configuration in the lowest-energy trion^34^. If $\tau _{\mathrm{r}}$ equals an integer number of the spin precession period (phase synchronization condition (PSC)), this mechanism leads to the spin saturation effect (SSE)^21^ where spin polarization is reduced as compared to the resonant spin amplification in a many-electron system^22,35,36^. As has been shown in ref. ^22^, this modification may lead to a spin mode-locked signal, but not because the distribution of spin precession frequencies is changed to a comb-like spectrum, but just because the spin polarization saturates differently for different precession frequencies. A second explanation^23^ considers a time-dependent Knight field that originates from the average transverse electron spin polarization and induces nuclear magnetic resonance. The trion-related SSE reduces the Knight field at PSC, hence locking the nuclear polarization. The third model^21^ considers optically stimulated fluctuations of the nuclear spin polarization. At PSC, optical excitation of trions is reduced, and because of the different Zeeman energy of electrons and nuclear spins, nuclear fluctuations are strongly suppressed, thus keeping the dot-ensemble in a mode-locked condition. A fourth explanation^24,25^ considers an optical Stark effect generated by detuned pump pulses that rotates the *S*_*y*_ spin component into the magnetic field direction and thereby generates DNP. This mechanism has been recently found to cause mode-locking in ensembles of single electron spins bound to impurities in ZnSe^20^.

In our many-electron dots the Fermi energy is large enough such that excitation of trions is screened and band-to-band transitions dominate^37^. In such a scenario, additional spin polarization can always be accommodated irrespective of previous polarization. This contrasts with the assumption in the first three explanations of spin mode-locking. In the following, we first investigate whether spin mode-locking has a purely electronic origin or whether it is related to nuclear spin polarization. We then develop a model that predicts mode-locking in case of DNP and SSE and compare it to our experimental observations. We finally test whether the optical Stark effect^38^ affects the observed spin mode-locking.

### Nuclear origin of mode-locking

**Fig. 2:**
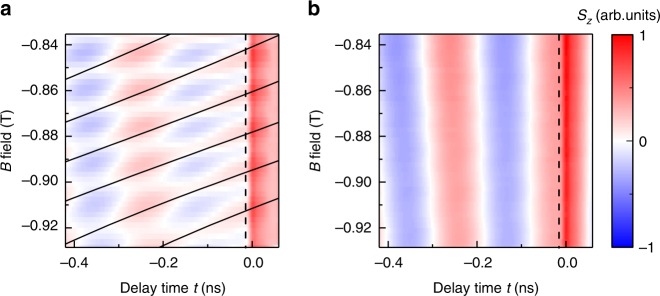
Dependence on nuclear polarization. **a** Recorded *S*_*z*_(*t*) of a dot array (400 nm) immediately after depolarization of nuclear spins. The magnetic field *B* is scanned from −0.93 to −0.83 T in 2.5 mT steps. Same normalization of *S*_*z*_ is used in (**a**, **b**). The black lines mark the expected positions of constant phase where *S*_*z*_ should attain maximum values in an unlocked spin system. **b** Signal from sixth scan, recorded 90 s after nuclear spins depolarization. The system is saturated and spin precession is mode-locked to the repetition rate of the laser, visible as a constant phase of the spin signal at *t* = −15 ps (dashed line)

We depolarize nuclear spins by leaving the sample in a zero external magnetic field and by blocking the laser pulses with a mechanical shutter. The system is kept in this condition for 3 min. We then ramp the magnetic field to a target field *B*, open the shutter, and immediately start to record six scans where for each scan *t* is swept from 50 to −430 ps within a laboratory time of 15 s. We repeat the same procedure for different *B* and obtain *S*_*z*_(*B*, *t*) for different laboratory times. Measurements are shown in Fig. 2 for a dot-ensemble (400 nm diameter) with 200 μW pump and 10 μW probe power. In the first scan (Fig. 2a), taken directly after nuclear spin depolarization, we observe that as a function of *B*, *S*_*z*_ oscillates about zero at *t* = −15 ps (dashed line). This is consistent with an unlocked spin system where the spin precession phase is given by $\varphi = \omega \cdot (t + \tau _r)$, with $\omega$ the angular precession frequency. We extract $\omega$ from oscillations in *S*_*z*_(*t*) at positive *t* and show the expected lines of maximum positive *S*_*z*_ (corresponding to *φ* = 2π*n*) at negative *t* as black lines in Fig. 2a. Down to −100 ps, the measured spin pattern indicates an essentially unlocked spin system. At more negative *t*, regions of constant *S*_*z*_ start to deviate from the expected lines of constant phase and instead align into bands of constant *t*, an indication for mode-locking. In the sixth scan (Fig. 2b), starting almost 80 s after nuclear spin initialization, the mode-locking is completed and is visible as a pure oscillation of *S*_*z*_ in *t* with a phase that is independent on *B*.

**Fig. 3:**
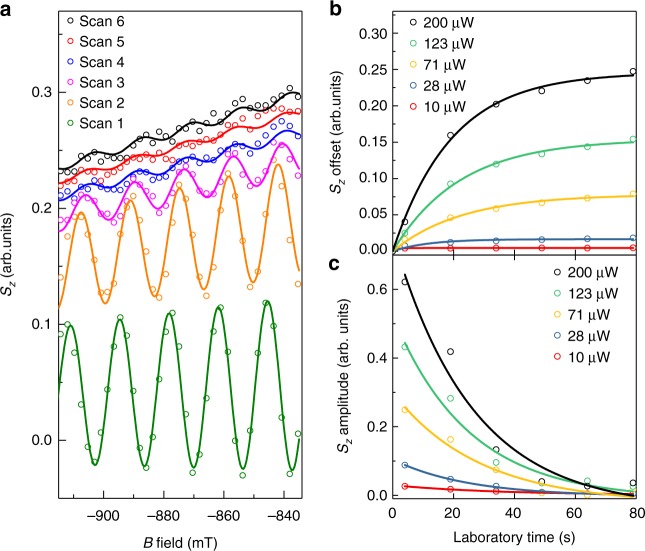
Build-up of mode-locking. **a** *B*-field dependent Kerr signal at *t* = −15 ps for six scans taken at different times after depolarization of the nuclear spin polarization (difference between each scan is 15 s). An increase in offset or decrease in amplitude is a measure for mode-locking. **b**, **c** Extracted offset and amplitude of the oscillations in *B* from the fit in (**a**). Data are shown for different pump powers and a fixed probe power of 10 µW. The characteristic offset build-up time or amplitude decay time is pump power independent and is extracted from the fit to be 25 s

The observed disappearance of spin mode-locking for depolarized nuclear spins indicates that the mechanism must originate from hyperfine interaction to nuclear spins. To extract the characteristic mode-locking build-up time *t*_ML_ in our system, we analyze traces of *S*_*z*_ taken at *t* = −15 ps (dashed lines in Fig. 2a, b). For an unlocked system, we expect *S*_*z*_(*B*) to oscillate about a zero-offset value. With a focusing of the spin precession frequencies onto PSC modes, the oscillation amplitude should decay and an offset should appear. Both the amplitude and the offset can be taken as a degree of spin mode-locking. The measured slices of *S*_*z*_(*B*) at *t* = −15 ps for all six scans are shown in Fig. 3a. Already the first trace shows a small offset, indicating that the process of mode-locking has already started. With increasing laboratory time (scan number), the oscillation amplitude decreases while the offset increases; hence, the system is developping into the mode-locked state. A fit of the amplitude and offset is shown versus laboratory time in Fig. 3b, c. In these fits, we take into account a linear decrease of the offset and oscillation amplitude with *B*, which we attribute to a *B*-dependent spin lifetime. From the laboratory -time dependence of the offset and amplitude, we extract *t*_ML_ to be in the order of 25 s, roughly independent of pump power (data for different pump power is shown in Fig. 3b, c). This is expected for DNP in a doped system where the time scale of DNP is given by the correlation time^39^. We exclude that only statistical nuclear fluctuations as proposed in ref. ^21^ are driving the mode-locking because we observe a decay time of spin mode-locking for a sample kept in the dark that is on the same order as the build-up time under illumination (shown in the Supplementary Note 4). This demonstrates that for our samples and measurement conditions, nuclear polarization is not frozen at PSC by bleaching of the optical excitation. We want to point out that DNP leads to a frequency change of the average electron spins by *ω*_n_, which is visible in the phase relation of individual scans (scan 1 to scan 6) in Fig. 3a. The phase of *S*_*z*_(*B*) at a fixed negative pump-probe delay is proportional to $(\omega + \omega _{\mathrm{n}}) \cdot (t + \tau _r) = \omega t + \varphi _{\mathrm{n}}$, so the change in phase can be attributed to a variation of $\varphi _{\mathrm{n}}$ with laboratory time, induced by DNP.

### Model for mode-locking mechanism

The finding that mode-locking also occurs in many-electron QDs with a purely nuclear origin asks for a new explanation of the effect. We first outline a model that predicts mode-locking based on DNP and SSE also in many-electron QDs. The model requires an asymmetry of spin pumping triggered by an oblique angle of the applied magnetic field and a finite average of circular polarization of the pump pulses. We then present data that suggest that the observed spin mode-locking does not completely disappear for zero average spin pumping and that a mechanism compatible with the optical Stark effect is involved.

**Fig. 4:**
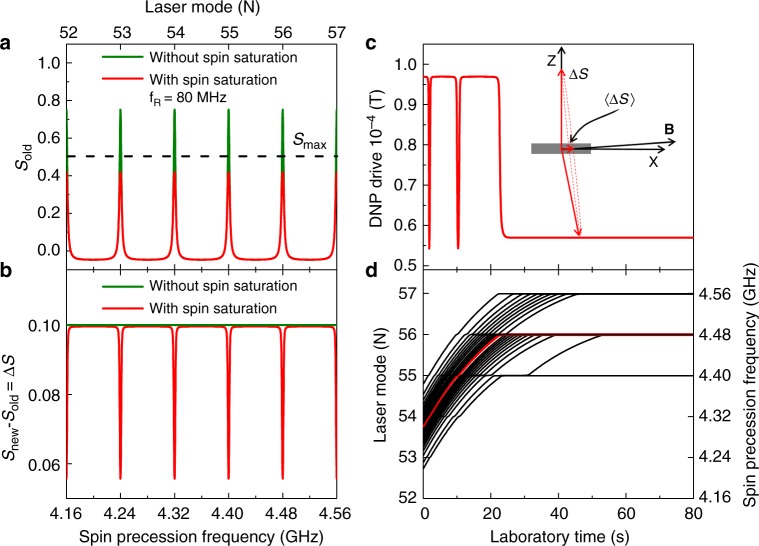
Model for spin mode-locking. **a** Calculated resonant spin amplification for an unsaturated spin system (green) and a saturated system (red). **b** Calculated spin excitation per laser pulse using Eq. (1) and assuming a spin polarization per pulse of *P* = 0.1 for saturated (red) and unsaturated (green) spin system. **c** Dynamical nuclear polarization drive $\langle {\boldsymbol{S}}\rangle {\mathbf{B}}$ calculated from the coupled Eqs. (2) and (3). The insert shows the added spin polarization per pulse, ${\mathrm{\Delta }}S = S_{{\mathrm {new}}} - S_{{\mathrm {old}}},$ with a non-zero average along **B**. **d** Black lines show evolution of the spin precession frequency for a distribution of initial start frequencies centered at 4.30 GHz (between 52nd and 55th laser repetition mode). Evolution of red marked frequency corresponds to the DNP drive shown in (**c**)

Similar to ref. ^22^, we assume that the added spin polarization per pulse, ${\mathrm{\Delta }}S = S_{{\mathrm{new}}} - S_{{\mathrm{old}}}$, depends on the spin polarization *S*_old_ before the pulse, but not because of resonant trion absorption, but because the polarization of the Fermi sea cannot exceed 100%. We model this by assuming Δ*S* to saturate with an exponential factor:

1 $${\mathrm{\Delta }}S = P(1 - {\mathrm{exp}}( - |S_{{\mathrm{max}}} - S_{{\mathrm{old}}}|{\mathrm{/}}P)),$$

whereby *P* is the unsaturated spin excitation per pump pulse. This ensures that *S*_new_ will always be smaller than *S*_max_ = ±$\frac{1}{2}$ (corresponding to 100% spin polarization, see Supplementary Note 3 for a graphical representation). Starting with *S*_old_ = 0 and summing over enough laser pulses, we numerically determine the saturated spin polarization *S*_old_ as a function of spin precession frequency. In Fig. 4a, the obtained *S*_old_ is compared to that from an unsaturated system (described by Δ*S* = *P*, equivalent to the standard resonant spin amplification result^22,36^). The unsaturated curve exceeds the maximum spin polarization of *S*_max_ = $\frac{1}{2}$ at PSC. The saturated curve remains always below *S*_max_, which goes parallel with a substantial dip in Δ*S* within a narrow range around PSC (see Fig. 4b). As we will see in the following, this dip leads to a decrease in DNP which steers the nuclear spin polarization to a stable situation where electron spins mode-lock.

The hyperfine interaction is responsible for an Overhauser field **B**_n_ that is proportional to the nuclear spin polarization. It also causes DNP that changes **B**_n_ proportional to the average electron spin polarization **S**. This leads to a feed-back mechanism that is described by the following coupled equations^39,40^:

2 $$\frac{{{\mathrm{d}}{\mathbf{S}}}}{{{\mathrm{d}}t}} = \frac{{{\mathbf{S}} - {\mathbf{S}}_0}}{{\mathrm{\tau }}} - \frac{{g{\mathrm{\mu }}_{\mathrm{B}}}}{\hbar }{\mathbf{S}} \times ({\mathbf{B}} + {\mathbf{B}}_{\mathrm{n}}),$$

3 $${\mathbf{B}}_{\mathrm{n}} = K\frac{{\langle {\mathbf{S}}\rangle \cdot ({\mathbf{B}} + {\mathrm{\alpha }}\langle {\mathbf{S}}\rangle )}}{{B^2}}{\mathbf{B}}.$$

The first term on the right side of Eq. (2) describes the electron spin relaxation, where **S**_0_ is the steady-state spin. The second term describes the electron spin precession in the total magnetic field, **B** + **B**_n_, at a frequency given by the electron g-factor *g* and the Bohr magneton *μ*_B_. The steady-state nuclear field **B**_n_ can be calculated by Eq. (3), whereby *K* is a material-dependent constant and α is the Knight field constant. In the case of even a small misalignment of the magnetic field direction to the sample plane, **S**_new_ − **S**_old_ has a finite component along **B**, leading to a DNP drive component $\langle {\mathbf{S}}\rangle \cdot {\mathbf{B}}$ that is proportional to ${\mathrm{\Delta }}$*Sτ*/*τ*_r_ (see sketch in Fig. 4c). The transverse components of **S** enter the drive term proportional to *α*$\langle {\mathbf{S}}\rangle ^2$ in Eq. (3), which can be neglected because in our experiments the Knight field α$\langle {\mathbf{S}}\rangle$ from such transverse components is expected to be well below 1 mT, much smaller than the applied field **B**. We numerically solve the coupled system by taking the SSE into account (for detailed discussion see the Methods section). The simulation result in Fig. 4c shows the time-dependent DNP drive component $\langle {\mathbf{S}}\rangle \cdot {\mathbf{B}}$ for a spin at an initial frequency (at **B**_n_ = 0) of 4.30 GHz. It goes through two minima and after a certain time approaches a plateau. To understand such a behavior, we perform calculations for a set of initial start frequencies, express the total frequencies in terms of the laser repetition rate, and plot them versus laboratory time in Fig. 4d. The time-dependent DNP drive in Fig. 4c corresponds to the red curve in Fig. 4d. We find that the minima in the DNP drive term coincide with the PSC, directly related to the narrow dips in Δ*S* at PSC (see Fig. 4b). This reduction of the DNP drive at PSC realigns the initially uniformly distributed electron spin precession frequencies into discrete PSC modes. This leads to spin mode-locking and occurs on a timescale given by the DNP process (evaluated as *t*_ML_ in the experiment). The spectral width of the dips in Δ*S* and therefore also of the PSC modes is proportional to the spin decay rate 1/*τ*. Importantly, this decay rate is sampled over the build-up time of spin polarization by the laser pulse train, i.e. at most a few *τ*. Similar to dynamic decoupling protocols and other methods^41^, but here in a self-driven way, this mechanism acts as a high-pass filter for nuclear fluctuations with a cut-off frequency at 1/*τ*, leading to a stabilization of nuclear polarization and a corresponding increase in electron spin lifetime.

**Fig. 5:**
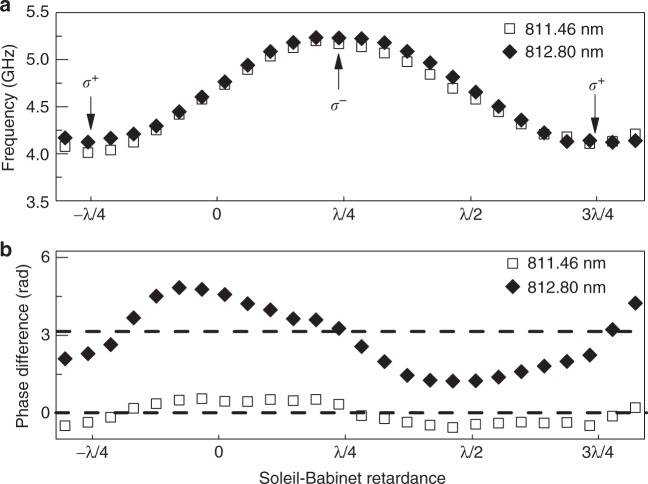
Helicity and wavelength dependence of the nuclear focusing. **a** Larmor precession frequency of electron spins for a pump wavelength of 811.46 and 812.80 nm as a function of the average pump helicity (set by an additional Soleil-Babinet compensator) at *B* = 0.93 T applied 8° out of the plane of the sample. Arrows with labels *σ*^+^ and *σ*^−^ indicate maxima of average pump helicity. At a retardance of 0, the polarization of the pump is equally modulated between *σ*^+^ and *σ*^−^. **b** Phase difference of spin precession at negative and positive time delay at *t* = 0 ps for pump excitation at 811.46 nm (mode-locking) and 812.80 nm (anti-mode-locking). Horizontal dashed lines indicate expected lines of constant phase for a mode-locked (phase difference 0) and anti-mode-locked (phase difference *π*) spin system

The proposed mechanism in the model works only if a finite component of spin polarization is injected along the magnetic field direction. This could occur in case of an (unintentional) misalignment of the magnetic field with respect to the sample plane, together with a non-zero average of the helicity of the pump photons. We therefore measure spin precession in the same sample but with a magnetic field that has been intentionally tilted out-of-plane by an angle of 8°. By adding a Soleil-Babinet (SB) variable retarder after the photoelastic modulator, we control the average helicity of the pump photons while still modulating the retardance at a peak-to-peak amplitude of *λ*/2 and a frequency of 50 kHz. For the SB set at retardance of 0 (*λ*/2), the pump photons are modulated equally between right- and left circular polarization with linear *p* (*s*) polarization in between. For a retardance of *λ*/4 (3*λ*/4), the pump photons are modulated between *p* and *s* linear polarization while going through *σ*^−^ (*σ*^+^) polarization. In an oblique field configuration, the retardance of *λ*/4 and 3*λ*/4 should provide the strongest positive and negative DNP, which we consistently observe as a maximum and minimum of the spin precession frequency (see Fig. 5a and Supplementary Note 6). The corresponding difference between the phases of the positive and negative delay oscillation of *S*_*z*_ is shown in Fig. 5b as squares. We see that for all settings of the SB, the phase difference is locked to a value around zero. At the position of maximum DNP, it goes through zero, whereas in between, phase offsets into opposite directions occur.

### Optical Stark effect

We finally test the role of an optical Stark effect induced by the pump photons. As has been discussed in refs. ^20,24,25^, pump pulses detuned from the optical transition of the dots could lead to spin rotations into the magnetic field direction. Interestingly, positive and negative detuning are expected to provide locking into modes that are either situated at integer multiples of the laser repetition rate, or in between those positions^20^. When we detune the pump wavelength from 811.46 to 812.80 nm, we find a sudden change of the phase difference (diamonds in Fig. 5b) that is now centered around *π* instead of 0. This corresponds to an anti-mode-locking situation where the precession frequency is focused in between integer multiples of the laser repetition rate. This observation suggests that the optical Stark effect plays a dominant role for mode-locking in our large dots with many electrons, even at oblique field configurations where the SSE contribution to DNP should be significant. Even more prominent than in the mode-locked case at *λ* = 811.46 nm, an SB retardance of 0 and *λ*/2 leads to maximum deviations of the phase difference from the mean value of *λ*, which underlines the importance of the exact polarization of the pump beam during the cycle of the 50 kHz modulation. Such deviations of the phase difference are not expected from the optical Stark effect alone and may indicate an interplay with another mechanism, possibly the SSE model discussed above. Note that anti-mode-locking is the result of nuclear focusing away from the narrow mode-locking frequencies, which may explain the larger deviations from the ideal phase difference in that case.

## Discussion

We have shown that the precession of localized electron spins in nano- and micrometer-sized structures can be synchronized to a periodic drive. This synchronization occurs because of a rearrangement of nuclear spin polarization in each dot. By a hyperfine-induced feed-back mechanism, the additional precession frequency from the nuclear polarization saturates, such that the total spin precession frequency is placed at a multiple of the laser repetition rate (mode-locking) or in between (anti-mode-locking). Because the feed-back depends on the electron spin polarization whose spectral features are narrowed by resonant spin amplification to a width ~1/*τ* ^35^, the distribution of the final precession frequency can be narrowed to a similar width. In this way, the electron coherence time in principle can overcome the limit given by a fluctuating nuclear background. This approach can be applied in general to all materials with nuclear background and a saturable spin system, thus in addition opening possibilities to synchronize electro-optical and opto-mechanical systems to localized spins. Here demonstrated using an optical pulse train, spin mode-locking can in principle be driven purely electrically or via piezoelectric effects also mechanically. The observed long electron spin lifetimes in many-electron QDs may also enable to couple such spins to microwave and phononic cavities, exploring the regime of cavity-quantum electrodynamics with localized spins^42–44^.

## Methods

### Sample fabrication

The sample is fabricated from a modulation-doped GaAs/AlGaAs quantum well structure which is grown by molecular beam epitaxy. The quantum well is 18 nm wide and is located 143 nm below the sample surface. The carrier density of the quantum well is $n_{2{\mathrm{D}}} = 2.15 \times 10^{11}{\kern 1pt} {\mathrm{cm}}^{ - 2}$ under illumination, and we measure an electron *g-* factor with absolute value of 0.372. For fabrication of a QD ensemble, we use positive resist for electron beam lithography. After resist developing, 150 nm of aluminum is evaporated. The aluminum layer serves in the next step as an etch mask for reactive ion etching with an HBr plasma. In this way the material around the deposited aluminum is removed (700 nm is etched from the surface). In the last step, the deposited aluminum is removed with KOH solution.

### Experimental settings

The system is investigated with degenerate pump-probe spectroscopy with ps laser pulses at a wavelength of 811.5 nm which is in resonance with the quantum well transition. The photoluminescence spectrum of the QD array is peaked at 811.5 nm with a full-width at half maximum of 1.4 nm (see Supplementary Note 5). The full-width at half maximum of the pump/probe pulses is 1.0 nm. All experiments are performed at 15 K. The laser repetition rate is 80 MHz. The focused laser spot size of pump and probe is 30 µm. The probe power is fixed for all experiments and is 10 µW. In the measurements shown in Fig. 1d and those in Fig. 1 of the Supplementary Note 2, a waiting time of 5 min before each scan of the pump-probe delay ensured saturation of the nuclear focusing.

### Data fitting

The Kerr signal in Fig. 1b is fitted with an oscillating exponential decay of the form $S_z(t) = A \cdot e^{ - {t/{\tau^{*}}}}{\mathrm{cos}}(\omega t + \varphi _0)$ for positive time delays. A lifetime $\tau ^ \ast$ of 6.8 ns is obtained for the data shown in Fig. 1b. The same fitting function was used to extract the phase information ($\varphi = \omega t + \varphi _0$) from the data shown in Fig. 1d. The fitting function is applied to a short time delay range at positive and negative time delays for each magnetic field *B*, and the extracted phase is shown in Fig. 1c.

### Spin dynamics model

Equation (2) can be solved analytically, and the solution is decomposed into two orthogonal components transverse to **B**. Both components are defined by exponentially decaying harmonic oscillations. Spin accumulation is calculated by propagating **S**_new_ to **S**_old_ over a time *τ*_r_ using Eq. (2) and then determining a new ${\mathbf{S}}_{{\mathrm{new}}} = {\mathbf{S}}_{{\mathrm{old}}} + {\mathrm{\Delta }}S{\hat{\mathbf z}}$ until a steady-state spin polarization is obtained. In case of no SSE, the resonant spin amplification equations are recovered^22,36^. In case of even a small misalignment of the magnetic field direction to the sample plane, the additional spin polarization ${\mathrm{\Delta }}S{\hat{\mathbf z}}$ has a finite component along **B**, leading to DNP. Such DNP is reduced by employing a modulation scheme for the helicity of the optical pump pulses, but in practice is difficult to cancel to zero. In the simulation, we assume a misalignment angle of 2° and an imbalance between left- and right-circular polarization of 5%. From Eq. (2) we obtain **S**, from which the target nuclear field defined in Eq. (3) is calculated. The actual nuclear field **B**_n_ is adapted in small steps towards the target field with the long time constant of DNP, in each step solving again Eq. (2) and calculating a new target field. We want to point out that we assume **B**_n_ to be aligned along **B**, which is a good approximation considering the small Knight field in GaAs. We use the parameters *gK* = 7.79 T ^39^, *τ* = 100 ns and *α* = 0.

## Supplementary information

Supplementary Information

## Data Availability

The data that support the findings of this study are available from the corresponding authors upon reasonable request.
