# Supplementary material for: Universal nuclear focusing of confined electron spins
Source: Nat Commun. 2019 Mar 7;10:1097. doi: 10.1038/s41467-019-08882-y (PMC6405744; doi:10.1038/s41467-019-08882-y)
Supplement: Supplementary file 1 — Supplementary Information [file 41467_2019_8882_MOESM1_ESM.pdf]

Supplementary Information:

Universal nuclear focusing of confined electron spins

Markmann et al.

Supplementary Note 1: Estimation of electron numbers in a single quantum dot

The number of electrons in each quantum dot is given by the dot size and the carrier density of the quantum well. From Hall measurements we extract the carrier density of the quantum well to be $n_{2D} = 2.15 \cdot 10^{11}\ cm^{-2}$ under illumination. For our estimation we neglect the depletion length (l_D) of the carriers at the edge of the quantum dot, assuming that the quantum dot diameter is much larger than l_D. The table below shows the calculated number of electrons in each dot.

Dot diameter (nm)	Number of electrons
1800	5478
1000	1690
600	608
400	270

Table 1 : **Dot carrier density**. Calculated number of electrons in a single quantum dot from the quantum well carrier density n_{2D}

Supplementary Note 2: Mode-locking on different dot diameters.

In addition to the data presented in the manuscript we show that spin mode-locking occurs for different dot diameters. Exemplary we show in Fig. 1(a) a scanning electron microscopy image of a 600 nm dot array. In Fig. 1(b)-(d) data on spin mode-locking is presented for dot diameters of 500, 1000 and 1800 nm. The data in Fig.1(b)-(d) is recorded in the same way as described in the manuscript. For the 1800 nm dot array we observe that at high B and negative time delays the spin signal becomes weak. Such a behavior is attributed to a reduced spin lifetime induced by the larger influence of spin-orbit dephasing, and to a magnetic-field dependent spin lifetime.

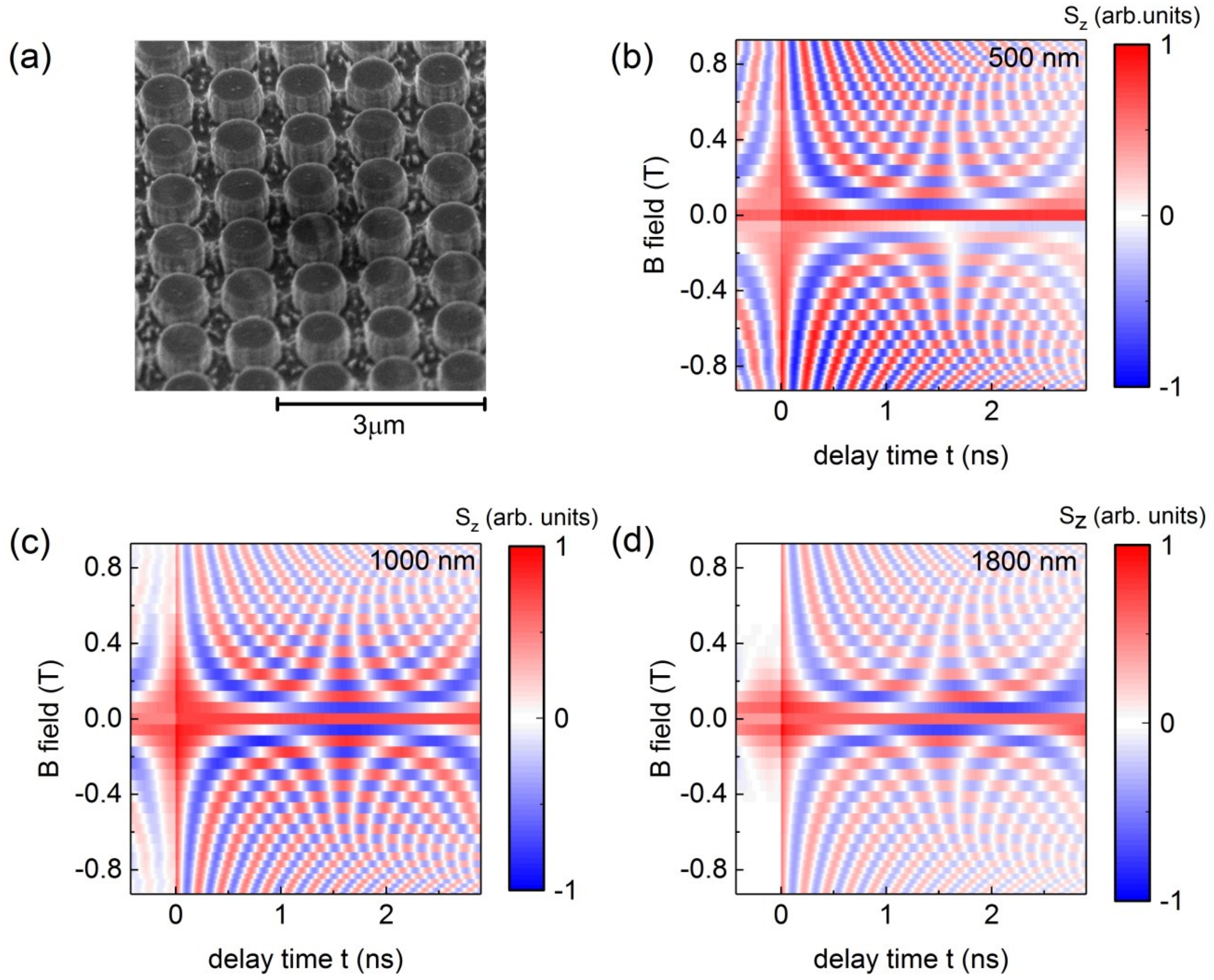

Supplementary Figure 1: **Spin mode-locking for different dot diameters**. (a) Scanning electron micrograph of lithographically defined quantum dots. (b)-(d) Time-resolved Kerr signals as a function of external magnetic field B for 500, 1000 and 1800 nm dot size arrays. The S_z signal is color coded and is normalized to +/- 1.

Supplementary Note 3: Model for spin saturation effect (SSE)

In order to account for the SSE, we define a spin dependent spin excitation function $S_{\text{new}}(S_{\text{old}})$. Equation (1) in the main text describes a heuristic function and is plotted in Fig. 2 (red curve) for an additional spin polarization per pulse of $P = 0.1$. For comparison, the saturation curve for singly-charged quantum dots with trion excitation is shown as a blue curve [1]. For trion excitation, the additional spin excitation per pulse depends linearly on S_{old}. In our model for a Fermi sea, it is constant unless $S_{\text{old}} + P$ becomes close to full polarization.

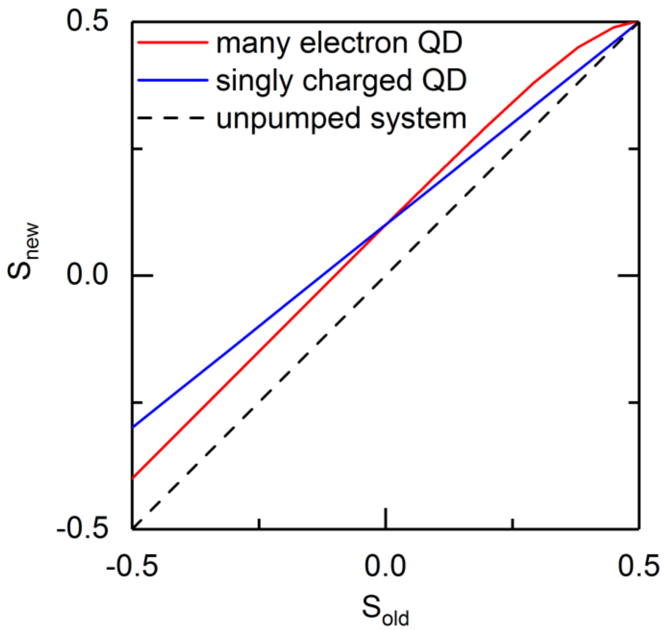

Supplementary Figure 2: **Spin saturation**. Spin dependent spin excitation for singly charged quantum dots and for many-electron quantum dots (red curve) as used in our model (spin polarization per pulse is $P = 0.1$).

Supplementary Note 4: Decay of spin mode-locking

We describe measurements of the characteristic decay time of spin mode-locking after the periodic spin excitation is switched off. For this, we first saturate the mode-locking by periodical optical excitation for 5 min in an applied external B field of -0.93 T. We then block the laser pulses with a mechanical shutter, wait for a certain time and after unblocking the laser record the Kerr signal for time delays between -0.43 ns to 2.8 ns. We repeat this procedure for waiting times t_{lab}(laboratory times) between 5 and 150 s. The obtained data is shown in Fig. 3 (a). For negative time delays, we observe a change in the Kerr signal phase with increasing laboratory time. Additionally, we observe a decrease of the Kerr signal amplitude at negative delay times. Both are due to depolarization of the nuclear spins, resulting in a decrease of the nuclear field from the value obtained by DNP, and in a broadening of the ensemble spin precession frequencies. This is in agreement with our model (see Fig. 4 (d) in the manuscript), showing that the Larmor precession frequencies are not only distributed into discrete modes, but their average also increases as compared to the depolarized case. We see the change in the average precession frequency also from the Kerr signal at positive time delays. The same time scale of the build-up and the decay of the Kerr signal indicates that this is due to spin-polarized conduction band electrons of the dot array hyperfine-coupled to the nuclear spins [2]. Figure 3(b) shows measured $S_z(t)$ at laboratory times 5 and 140 s. The two signals oscillate at different frequencies, which we extract by fitting oscillating decaying curves for positive time delays. The frequency in the mode-locked case (5 s laboratory time) is 22 MHz higher than in the partially locked case (140 s laboratory time). While taking the measurement of $S_z(t)$, the laser pulses drive spin mode-locking, thus the fitted Larmor precession frequency at 140 s is slightly overestimated. A dynamical frequency shift is also

reflected in our model [Fig. 4 (d) in the main text]. The initial Larmor precession frequencies (without DNP at laboratory time 0 s) shift to a higher frequency once the DNP sets in.

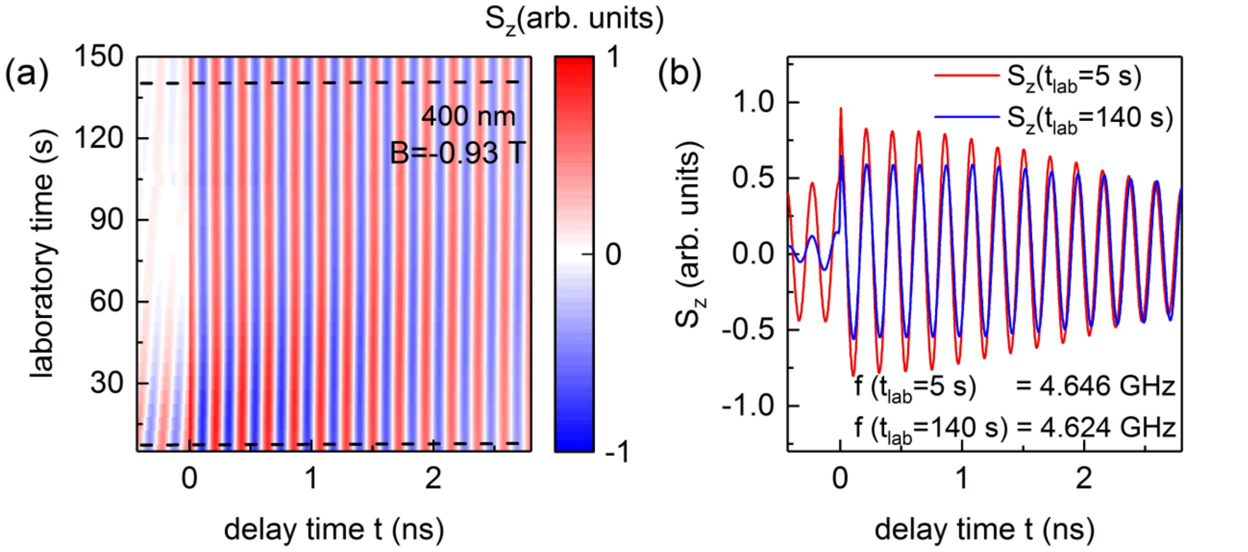

Supplementary Figure 3: **Mode-locking decay**. (a) Decay of spin mode-locking studied in 400 nm dot array at B = -0.93T. The Kerr signal (measuring the spin component S_z) is color coded and is normalized to +/- 1. (b) Slices of (a) which are indicated with dashed lines. Kerr signal at laboratory time 5 s (red curve) and 140 s (blue curve) exhibit different precession frequencies at positive time delays, indicative for a decay of the DNP.

Supplementary Note 5: Optical characterization of the investigated dot array

A photoluminescence spectrum is recorded of the 400 nm dot array by exciting the sample with a linearly polarized laser at 735 nm. The luminescence of the dot array is shown in Fig 4(b). The maximum of the photoluminescence emission is at 811.5 nm with a full-width at half maximum of 1.43 nm. For comparison, a spectrum of the pump/probe laser is shown in Fig. 4(a).

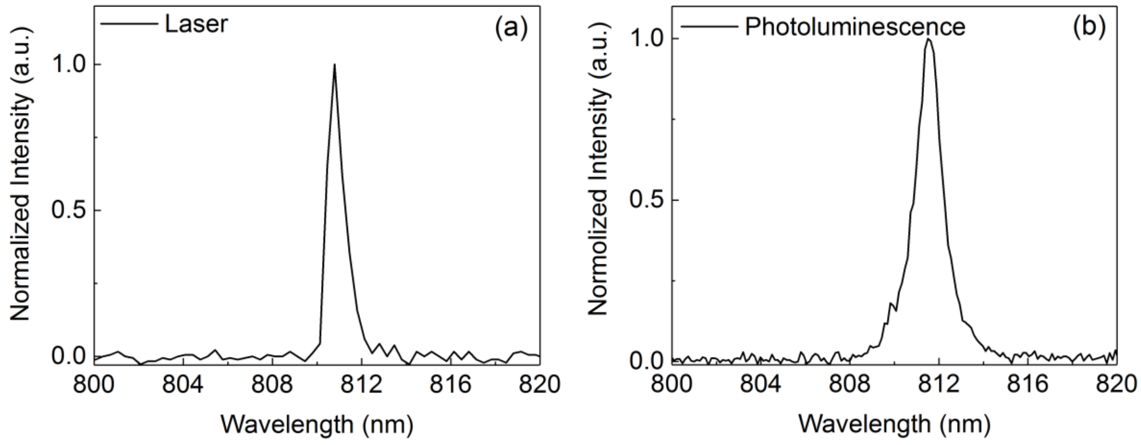

Supplementary Figure 4: (a) spectrum of the pump/probe laser centered around 811 nm. (b) photoluminescence spectrum of the 400 nm dot array.

Supplementary Note 6: Helicity dependent Kerr spectroscopy

Helicity dependent measurements at wavelengths of 811.46 nm and 812.80 nm are performed at a constant magnetic field B = 0.93 T applied at an out-of-plane angle of 8 degrees.

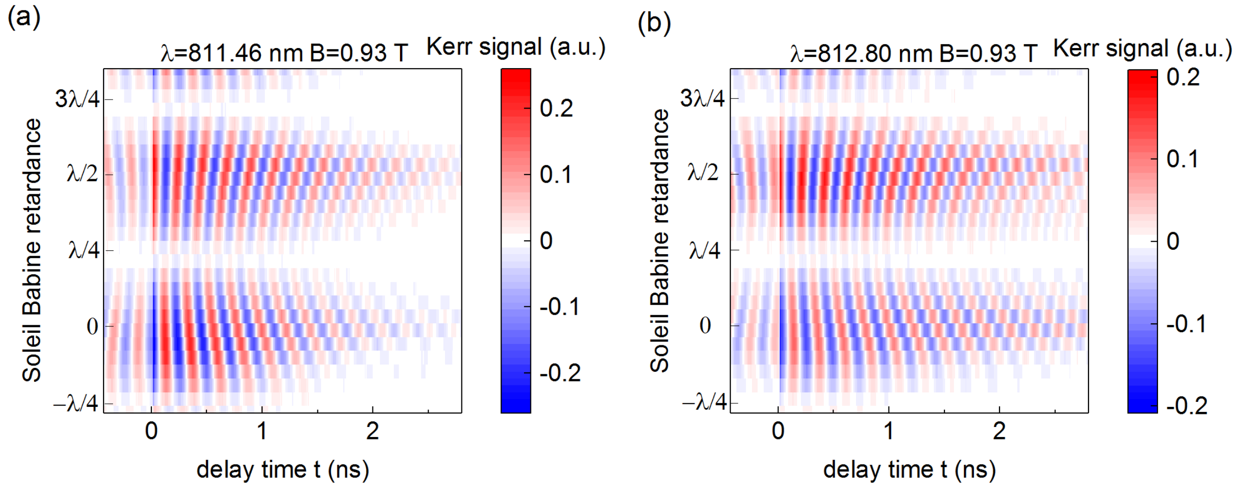

Supplementary Figure 5: (a)/(b) Time-resolved Kerr signal of the dot array at B = 0.93 T as a function of pump-probe delay time and a Soleil Babinet retardance for 811.46 and 812.80 nm. For a retardance of 0 and λ/2, the pump helicity is modulated between σ^+ and σ^- providing maximum Kerr rotation signal. At retardance of λ/4 or 3λ/4, the pump helicity is modulated between p and s linear polarization while going through σ^- or σ^+ in between. This leads to a disappearance of the Kerr rotation signal as detected by the lock-in amplifier, but at the same time leads to a maximum or minimum in the spin precession frequency because of maximum DNP.

Supplementary References

1. Yugova, I. A., Glazov, M. M., Yakovlev, D. R., Sokolova, A. A. & Bayer, M. Coherent spin dynamics of electrons and holes in semiconductor quantum wells and quantum dots under periodical optical excitation: Resonant spin amplification versus spin mode locking. *Phys. Rev. B* **85,** 125304 (2012).
2. Korringa, J. Nuclear magnetic relaxation and resonance line shift in metals. *Physica* **16**, 601 (1950).
